# Ruthenium Complexes as Promising Candidates against Lung Cancer

**DOI:** 10.3390/molecules26154389

**Published:** 2021-07-21

**Authors:** Qi Sun, Yingsi Li, Hongdong Shi, Yi Wang, Jitian Zhang, Qianling Zhang

**Affiliations:** 1 International Cancer Center, Guangdong Key Laboratory for Genome Stability & Disease Prevention, Department of Pharmacology, Shenzhen University Health Science Center, Shenzhen 518060, China; sunqi@szu.edu.cn (Q.S.); LiYingsi2@126.com (Y.L.); 2 College of Chemistry and Environmental Engineering, Shenzhen University, Shenzhen 518060, China; shongd@szu.edu.cn; 3 Key Laboratory for Advanced Materials of MOE, School of Chemistry & Molecular Engineering, East China University of Science and Technology, Shanghai 200237, China; wangyi0435@sina.com; 4 Department of Surgery, The University of Hong Kong-Shenzhen Hospital, Haiyuan 1st Road, Futian District, Shenzhen 518053, China

**Keywords:** Ruthenium complexes, lung cancer

## Abstract

Lung cancer is one of the most common malignancies with the highest mortality rate and the second-highest incidence rate after breast cancer, posing a serious threat to human health. The accidental discovery of the antitumor properties of cisplatin in the early 1960s aroused a growing interest in metal-based compounds for cancer treatment. However, the clinical application of cisplatin is limited by serious side effects and drug resistance. Therefore, other transition metal complexes have been developed for the treatment of different malignant cancers. Among them, Ru(II/III)-based complexes have emerged as promising anticancer drug candidates due to their potential anticancer properties and selective cytotoxic activity. In this review, we summarized the latest developments of Ru(II/III) complexes against lung cancer, focusing mainly on the mechanisms of their biological activities, including induction of apoptosis, necroptosis, autophagy, cell cycle arrest, inhibition of cell proliferation, and invasion and metastasis of lung cancer cells.

## 1. Introduction

Primary bronchogenic carcinoma, also known as lung cancer, is a malignant tumor that originates from the bronchial mucosa or gland. With a high incidence and mortality rate, lung cancer poses a serious threat to human health, while its cases and deaths rise every year [1]. Although its incidence follows breast cancer, lung cancer remains the leading cause of cancer death, with approximately 1.8 million deaths (18%) worldwide [2]. Moreover, the global cancer burden is expected to increase by 47% in 2040 compared to 2020, reaching a total of 28.4 million cases [2]. However, current advances in novel chemotherapeutic agents, targeted therapies, standardized diagnosis, and staging and multidisciplinary treatment of lung cancer have improved patient survival rates [3]. Nevertheless, the prognosis of lung cancer patients is still poor due to insufficient early diagnosis.

Lung cancer can be classified into central and peripheral lung cancer depending on the anatomical part affected, as well as into two main pathological entities: non-small-cell lung cancer (NSCLC) and small-cell lung cancer (SCLC). NSCLC can be further subdivided into three histological subtypes: lung squamous cell carcinoma, lung adenocarcinoma, and large cell carcinoma. NSCLC, which accounts for about approximately 85% of lung cancer cases, has shown an increased mortality rate in recent years. Radical surgery is the most common treatment applied to early-stage NSCLC patients, while chemotherapy is mainly used for NSCLC patients in advanced or recurrent stages [3]. Moreover, NSCLC patients with unresectable tumors in the advanced stage are still treated with Pt-based doublet chemotherapies such as cisplatin–etoposide and carboplatin–paclitaxel [4]. Currently, chemotherapy combined with Pt-based antineoplastic agents, such as cisplatin, oxaliplatin, and carboplatin, has been efficiently used to treat various cancers, including NSCLC. However, lung cancer patients show different sensitivity to Pt-based chemotherapy and 20–40% of them tend to relapse within six months after treatment [5]. It is also known that the anticancer activity of cisplatin targeting nuclear DNA is based on the formation of cisplatin–DNA adducts, which stop DNA replication and transcription, while triggering cancer cell apoptosis [6,7]. However, tumor resistance to cisplatin reduces the accumulation of drugs in cancer cells, rapid DNA repair, and upregulation of transcription factors [8], thus significantly limiting its clinical application. Moreover, Pt drugs can lead to serious side effects, such as nephrotoxicity, ototoxicity, nausea, vomiting, hair loss, etc., further limiting their effective use [8,9]. Therefore, researchers have focused on the development of alternative anticancer drugs to overcome the drawbacks of Pt-based agents in NSCLC patients.

Considering the effectiveness of cisplatin and its derivatives, other transition-metal complexes, such as Ru-, Ir-, Rh-, Pd-, Au-, and Os-based complexes, have emerged as a new generation of promising anticancer agents due to their potential anticancer properties and selective cytotoxic activity [10,11,12,13]. Among them, Ru complexes have received particular attention owing to their good biodistribution and multimodal actions. Moreover, Ru compounds can effectively bind to the serum transferrin receptor, which is highly expressed in tumor cells, thus increasing the number of Ru–transferrin complexes that could preferably be delivered at the tumor site [14,15]. Ru can be found in two stable oxidation states (II and III) [16], which can coordinate with ancillary ligands of different geometries to prepare diverse Ru(II/III) complexes with different steric and electronic properties [17]. For example, arene has been widely used as a ligand, as it can stabilize the oxidation state of metal complexes. Hence, a series of hydrophilic and hydrophobic arene Ru(II/III) complexes have been designed and synthesized with great potential for the development of metal-based chemotherapeutic drugs [18,19]. There are few studies focused on Ru(IV) complexes that search for efficient anticancer candidates. Of note are the recent research reported by Lu, Y et al. who have proposed a novel dual-targeting Ru(IV) candidate with antitumor and antimetastatic properties in vitro and in vivo studies via the PARP/ATM pathway [20]. There are different signaling pathways that participate in the anticancer activity of various Ru complexes, including the mitochondria-mediated pathway, the DNA damage-mediated pathway, and the death receptor-mediated pathway [21,22].

**Figure 1:**
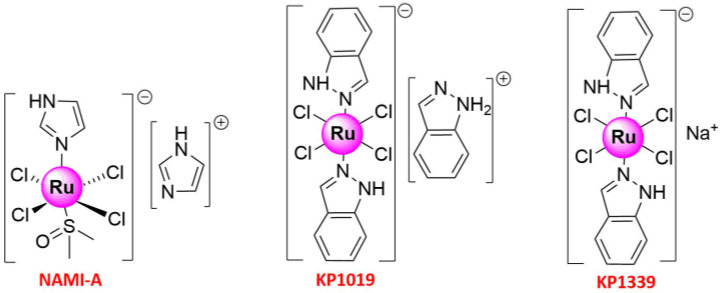
Structures of three important Ruthenium complexes entering clinical trials.

Ru complexes can also trigger phototoxicity in cells, which induces a series of photochemical and photobiologic processes, leading to irreversible photodamage in tumor tissues [16]. Thus, Ru compounds, such as Ru(II) polypyridyl complexes, which could interact with bovine serum albumin (BSA) and with DNA via minor grooves [23,24,25,26], are considered attractive photo-mediated activation prodrugs for photodynamic therapy (PDT) and photoactivated chemotherapy (PACT) [27]. Given also that photofrin, a hematoporphyrin derivative, is the only PDT drug approved by the Food and Drug Administration for cancer therapy, including early and advanced lung cancer [28,29], extensive studies have been performed to develop novel Ru(II/III) complexes for efficient cancer treatment [16,18,30]. Metal-based anticancer candidate imidazolium [*trans*-RuCl_4_(1*H*-imidazole) (DMSO-S) (**NAMI-A**; DMSO = dimethyl sulfoxide) (Figure 1) was the first Ru compound to be studied on human beings, which has reached the phase II stage [31,32,33,34]. The study was launched in 2008 when **NAMI-A** administered in combination with gemcitabine was given to patients with advanced NSCLC [34]. Previously, fundamental works have shown that its lower molar cytotoxicity over cisplatin results from its reduced reactivity against DNA in intact cells, and more studies in animal models have exhibited the excellent and selective activity against lung metastases of some solid metastasizing tumors at a concentration with relatively mild toxicity [32,33,35]. The other two important promising Ru(III) complexes that have entered clinical trials were indazolium [*trans*-RuCl_4_(1*H*-indazole)_2_] (**KP1019**) [36,37,38,39] and sodium [*trans*-RuCl_4_(1H-indazole)_2_] (**KP1339**) [40] (Figure 1). The pharmacokinetics of **KP1019**, which was characterized by a small volume of distribution, low clearance, and long half-life, has been researched in a phase I dose-escalation study, in which five out of six patients treated with **KP1019** experienced disease stabilization with no severe side effects [36,39]. With these pioneering works, Ru complexes are attracting increasing attention from chemical researchers. The recent years have witnessed the development of Ruthenium complexes as second-generation metal-based anticancer agents, possessing high potency of targeting cancer cells due to their low toxicity, the ability to induce apoptosis, selective anti-invasion, and anti-metastasis activity [41,42,43]. Some of them have also shown anti-angiogenic properties and could therefore be used to inhibit angiogenesis, the basis of tumor growth and metastasis [44]. Moreover, with the purpose of improving their in vivo stability, solubility, cellular uptake, and effectiveness, some research groups with meticulous design have developed special drug delivery systems (nanoparticles, liposomes, etc.), which could encapsulate Ru-based compounds appropriately [30,45,46,47].

**Figure 2:**
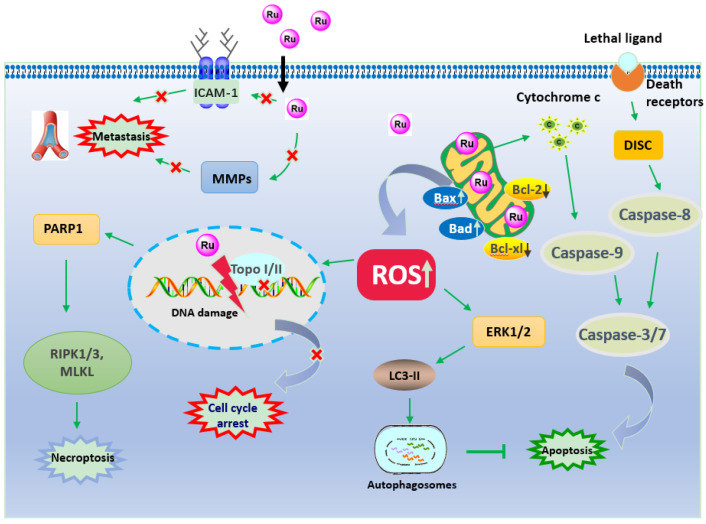
The mechanism of Ruthenium complexes against lung cancer.

Based on these promising results, in this review, we summarize the recent findings on the anticancer mechanisms of Ru-based compounds targeting lung cancer, including apoptosis, autophagy, necroptosis, anti-metastasis, and cell cycle arrest (Figure 2 and Table 1).

## 2. Ru(III) Complexes

Ru(III) complexes are rapidly evolving into next-generation anticancer drugs. Ru(III) hydrazone complexes owing uncoordinated phenolic oxygen were offered as better drug candidates than Ru(II) complexes with the same ligands because the phenolic oxygen in Ru(III) complexes could effectively interact with biomolecules by hydrogen bond, while phenolic oxygen of Ru(II) complexes was deprotonated for coordinating with Ru metal [50]. Moreover, several Ru(III) complexes that have entered clinical trials in different phases, such as **KP1019** [36,37,38,39], **KP-1339** [40], and **NAMI-A** [31,32,33,65], showed promising anticancer activity with limited side effects and have been used to prepare various derivatives.

### 2.1. Apoptosis

Recent studies have shown that Ru(III) complexes inhibit tumor cell proliferation by inducing apoptosis, which refers to genetically identified programmed cell death and plays a vital role in normal tissue homeostasis [66]. Apoptosis is not only involved in the occurrence and growth of tumors but can also trigger cancer cell death, making it a suitable pathway for the development of anticancer drugs [67]. There are two main types of apoptosis pathways: the intrinsic pathway, which depends on mitochondria, and the extrinsic pathway, which depends on death receptors [67,68].

**Figure 3:**
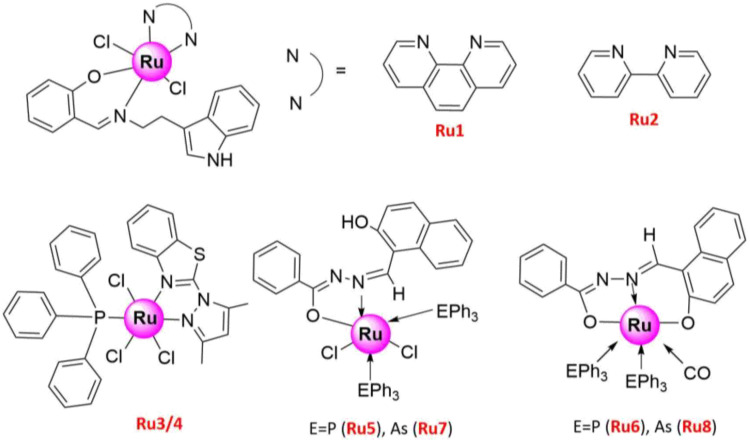
Structures of promising Ru(III) complexes inducing cell apoptosis.

The tryptamine-based mixed ligand Schiff base Ru(III) complexes Ru(Cl)_2_(SB) (phen = 1,10-Phenenthroline) (SB = Schiff base) (**Ru1**) and Ru(Cl)_2_(SB)(bipy) (bipy = 4,4′-bipyridine) (**Ru2**) (Figure 3) showed moderate antibacterial activity against Gram-positive and Gram-negative strains, but significantly high anticancer activity against NSCLC cells (H1299). In addition, they exhibited high cytotoxicity to NSCLC cells with IC_50_ = 10–12.5 ± 0.5 and 15–20 ± 0.5 μg/mL, respectively, but low toxicity to human erythrocytes compared to cisplatin [48]. Further cell studies indicated that the mechanism of action of Ru(III) complexes bearing tetradentate bis (aminophenolate) ligands against various cell types, including lung cancer, was based on the induction of programmed cell death [69]. In the case of *mer*- [RuCl_3_(PPh_3_)(dmpbt)] (**Ru3**) and *fac*- [RuCl_3_(PPh_3_)(dmpbt)] (**Ru4**) (PPh_3_ = triphenylphosphine, dmpbt = 2-(3,5-dimethylpyrazoll-yl) benzothiazole) (Figure 3), apoptosis was further promoted by their caspase 3/7 activity [49]. In another study, Ru(II) and Ru(III) hydrazone complexes {[Ru^III^(HL) Cl_2_(PPh_3_)_2_] (**Ru5**), [Ru^II^(L)(CO)(PPh_3_)_2_] (**Ru6**), ([Ru^III^(HL) Cl_2_(AsPh_3_)_2_] (**Ru7**), and [Ru^II^(L)(CO)(AsPh_3_)_2_] (**Ru8**)} (Figure 3) were prepared through a one-pot process and enhanced the release of lactate dehydrogenase, nitric oxide, and ROS [50]. The chromatin condensation, nuclear shrinkage, and plasma membrane blebbing were also observed using fluorescence microscopy, indicating that their in vitro cytotoxicity toward A549 cells resulted from apoptosis induction [50]. 

### 2.2. Anti-Metastasis

**Figure 4:**
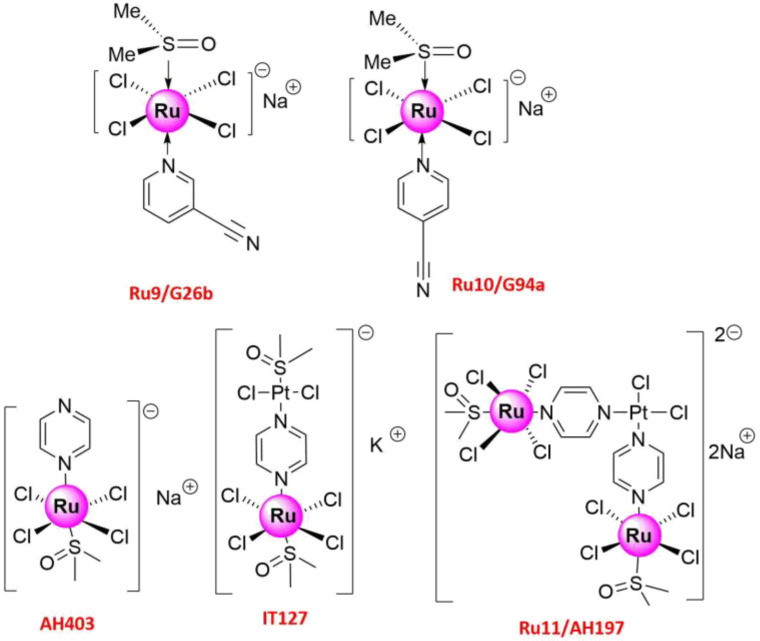
Structures of promising Ru(III) complexes inhibiting tumor metastasis.

Cancer therapies generally fail when genetically unstable cancer cells adapt to the tissue microenvironment, leading to tumor metastasis [70,71]. Tumor metastasis involves several steps, including loss of cell adhesion, increased motility and invasiveness, entry and survival into the circulation, and eventual settlement into new tissues or distant organs [72]. Metastatic tumors are resistant to various cancer therapies, especially drugs and radiotherapy [73], indicating the need for the development of new compounds targeting the tumor metastasis pathway [74]. To date, various analogs have been designed and synthesized to prevent metastasis and/or inhibit the growth of metastatic tumors. Antineoplastic Ru(III) complexes are currently the most effective, such as **NAMI-A**, which can greatly reduce lung metastasis and the formation of solid metastasizing tumors in mice [75]. Therefore, a series of **NAMI-A** derivatives have been prepared that could modify important metastasis parameters such as tumor invasion, matrix metalloproteinases (MMP) activity, and cell cycle progression [42]. The new analogs also maintained the potent characteristics of **NAMI-A** and could selectively interact with solid metastatic tumors. Furthermore, the introduction of different ligands improved their stability in aqueous solutions [76]. Two **NAMI-A** derivatives bearing a pyridine ligand, **Ru9/G26b** and **Ru10/G94a** (Figure 4), displayed little direct cytotoxicity to human (A549) and mouse Lewis lung cancer cells but had a significant suppressive effect on the invasion and migration of cancer cells [51]. Like with **NAMI-A**, in vivo studies in 4T1 mammary carcinoma-bearing mice showed that the occurrence and development of lung metastasis were suppressed significantly and that no retinal toxicity or hepatotoxicity was found in mice after intraperitoneal injection of **Ru9** and **Ru10** at a dose of 17.5 mg/kg per day for consecutive 6 days, with three times in a 1-day interval. **Ru9** in particular could suppress important molecules involved in metastasis, such as MMP-2 and MMP-9, and the vascular endothelial growth factor [51]. More interestingly, plasma atomic emission spectrometry showed that **Ru9** possessed higher metabolic stability due to having a longer Ru-elimination time in the lungs, indicating its better anti-metastatic effect compared to **NAMI-A** and **Ru10** [51]. In an earlier study, the hetero multinuclear complex [Na_2_] {[RuCl_4_(DMSO-S)(*μ*-pyz)]_2_PtCl_2_}, AH197 (**Ru11/AH197**) was also synthesized [77], and its effect on the motility and DNA electrophoretic mobility of NSCLC (A549) and breast cancer (MDA-MB-231) cells was compared to that of [K] [RuCl_4_(DMSO-S)(*μ*-pyz)Pt(DMSO-S)Cl_2_] (**IT127**), **NAMI-A**, and Na [*trans*-RuCl_4_(DMSO)(pyz)] (**AH403**) [78]. The Ru_2_Pt trinuclear species showed higher inhibitory activity in the order **Ru11** > **IT127** > **NAMI-A** > **AH403** (Figure 4) [78], while it was shown that the inhibition of cell motility might contribute to the anti-metastatic properties of the complexes [79]. 

## 3. Ru(II) Complexes

Ru(II) complexes are also known for their low toxicity, different modes of action, and non-cross resistance to traditional Pt-based drugs, especially cisplatin. Therefore, recent studies have focused on understanding the anticancer mechanism of Ru(II) complexes to develop more effective Ru(II)-based drug candidates.

### 3.1. Apoptosis

**Figure 5:**
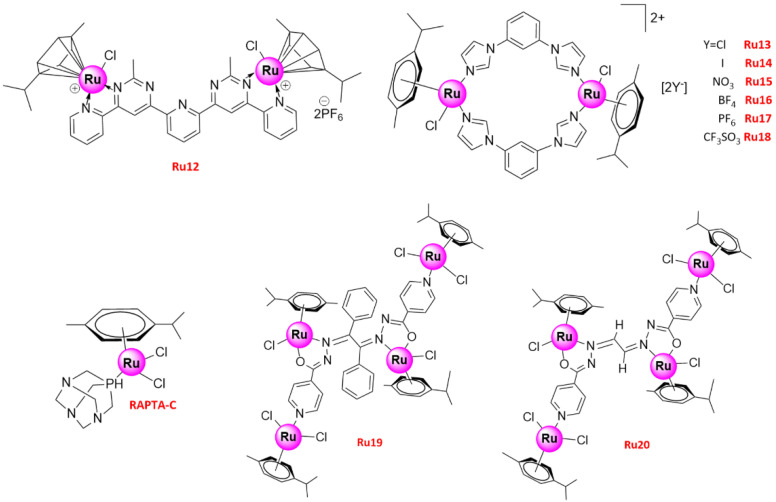
Structures of promising Ru(II) arene complexes inducing cell apoptosis.

With air stability, aqueous solubility, and structural diversity, the versatile half-sandwich Ru(II)-η^6^-*p*-cymene complexes have been reported as potential anticancer drugs, as they show distinct anti-proliferative activity and can effectively induce apoptosis [80]. For instance, [(Ru(η^6^-*p*-cymene)Cl)_2_(1,3,5-triaza-7-phosphaadamantane)] (**RAPTA-C**) (Figure 5) inhibited tumor metastasis and growth by inducing the apoptosis of Ehrlich ascites carcinoma cells through mitochondrial and p53-JNK pathways [81]. Similarly to **RAPTA-C**, a bimetallic Ru(II) cymene complex, [(Ru(η^6^-*p*-cymene) Cl)_2_(1,3-bis(2-methyl-6-(pyridin-2-yl) pyrimidin-4-yl) benzene)] (**Ru12**) (Figure 5), showed strong anticancer activity toward human NSCLC A549 and A427 cancer cells by inhibiting cell proliferation, migration, and invasion, which was stable in solution state in D_2_O/DMSO-*d_6_* mixture as well as in solid state under air and light [82]. Mitochondria-mediated apoptosis of NSCLC was also observed upon treatment with **Ru12**, followed by an increase in the apoptosis regulator Bax and caspase-3/-9 activation. Thus, **Ru12** induced DNA damage and cell death via caspase-dependent apoptosis by activating poly(ADP-ribose) polymerase(PARP) and triggering the p53-dependent pathway [82]. Moreover, **Ru12** inhibited cancer cell migration and invasion, which in turn blocked the expression of the c-Myc(myelocytomatosis) oncogene that is important for cell cycle progression, apoptosis, and cellular transformation, and it has been related to cancer metastasis [82,83]. In a current study, another two sets of organometallic arene Ru(II) complexes against cancer cells (NSCLC A549, colon adenocarcinoma LoVo, and hepato cellular carcinoma HuH-7) have been reported by Balaji, S and coworkers. Structurally, the complexes with *p*-cymene moiety outperformed the anticancer activity of the complexes containing benzene moiety, in that the latter had less hydrophobic interaction with the cell membrane [84].

Recently, six new bimetallic Ru(II) arene complexes [Ru_2_(η^6^-*p*-cymene)_2_(1,3-bib)_2_Cl_2_]X_2_ (X = Cl^−^ (**Ru13**), I^−^ (**Ru14**), NO_3_^−^ (**Ru15**), BF_4_^−^ (**Ru16**), PF_6_^−^ (**Ru17**), and CF_3_SO_3_^−^ (**Ru18**); 1,3-bib = 1,3-di(1*H*-imidazol-1-yl) benzene) were designed and synthesized [85]. **Ru13–18** (Figure 5) performed good stability in aqueous solution, and UV-Vis spectra also suggested that bidentate imidazole-based ligand strengthens the stability of the Ru-arene complex, comparing to mononuclear N-heterocyclic ligand. All showed moderate proliferative or anti-proliferative activity due to their interaction with glutathione on lung (A549) cancer cells, but strong intercalative binding ability to ctDNA. Moreover, **Ru14** showed a relatively better anti-proliferative activity compared to the other complexes due to the increased polarization of I^−^. Further treatment of A549 cells with **Ru14** led to concentration-dependent late apoptosis and cell cycle arrest in the G1/G0 phase. In a subsequent study, a hydrazone moiety was rationally incorporated into two tetranuclear arene Ru(II) complexes, **Ru19** and **Ru20** (Figure 5), to improve their pharmacological activity [86]. With high stability in DMEM containing 10%FBS and PBS with 10%DMSO, both complexes had high anticancer activity in vitro and could induce the apoptosis of various human cancer cell lines, including cisplatin-resistant lung (A549) cancer cells. To certify the systemic toxicity of **Ru19**, a series of animal experiments were conducted by the group. Impressively, no damage was observed in major organs, including the kidney, in the mice treatment with **Ru19** (6 mg/kg), while a mass of vacuolization in the cell cytoplasm of renal tubules were found in the cisplatin-treated mice, suggesting that the compound **Ru19** exhibited lower systemic toxicity and was potentially more tolerated by animals than cisplatin [86]. Apart from targeting cisplatin-resistant cancer cells to address chemoresistance issue, Teixeira, R. G et al. proposed a novel approach that organometallic Ru(II) compounds increased cisplatin cytotoxicity up to 1390-fold at nontoxic doses by inhibiting multidrug resistance-associated protein 1 (MRP1) and the P-glycoprotein 1 (Pgp) transporters [87]. It further promoted Ru(II) compounds as more valuable and prospective agents for lung cancer chemotherapy, in particular for those patients with cisplatin resistance.

**Figure 6:**
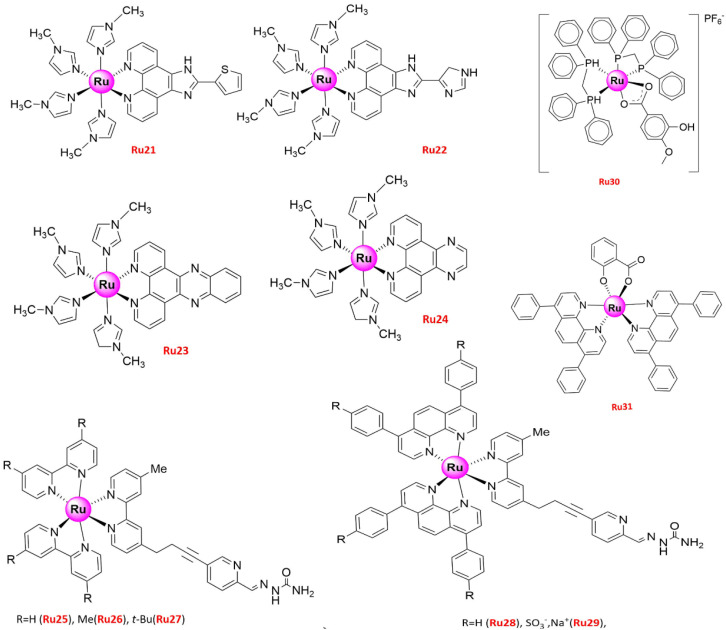
Structures of promising Ru(II) complexes inducing cell apoptosis.

A series of Ru(II) methylimidazole complexes (**Ru21–24**) (Figure 6) with strong anti-proliferative activity against various human cancer cells were also synthesized [88]. Although **Ru12** induced NSCLC apoptosis via the mitochondria-dependent pathway [82], two pathways were involved in the mechanism of action of **Ru21**. Specifically, **Ru21** activated the BID protein and depleted the mitochondrial membrane potential in A549 cells by regulating the expression of pro-survival and pro-apoptotic Bcl-2 family proteins [88]. Moreover, the Ru(II) polypyridyl complexes **Ru25** and **Ru28** (Figure 6) showed completely different mechanisms [52]. **Ru25** induced marginal oxidative stress and preferably accumulated in lysosomes, triggering apoptosis via an intrinsic mitochondrial pathway, while enlarged mitochondria were detected in **Ru25**-treated A549 cells. In contrast, **Ru28**, which was mainly localized in mitochondria and endoplasmic reticulum, did not have the same effect and induced caspase-independent apoptosis [52].

**Figure 7:**
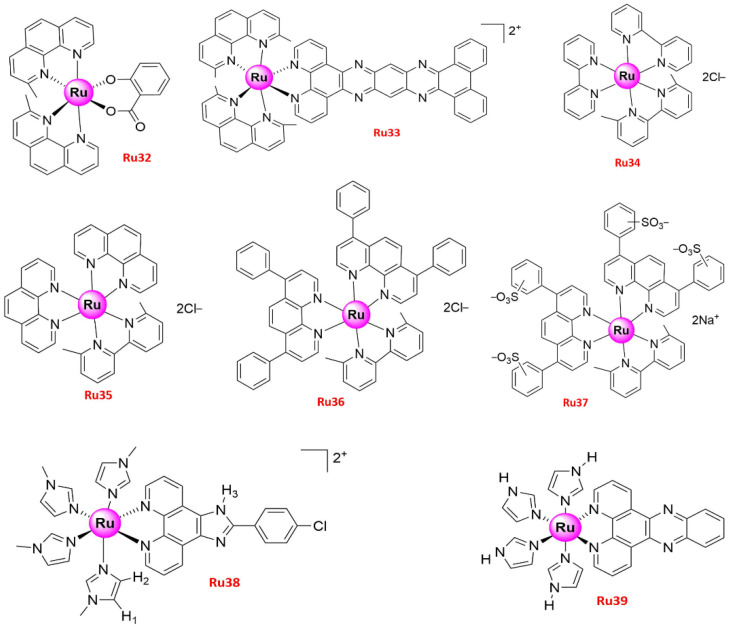
Structures of promising Ru(II) complexes inducing cell apoptosis and autophagy.

ROS are generated under normal cell activity and are involved in cellular signaling. However, high ROS levels produced mainly by mitochondrial dysfunction may lead to oxidative damage of cellular structures such as DNA damage and apoptosis [89,90]. Therefore, agents modulating ROS generation have been designed for clinical cancer therapy. For instance, **Ru30** [54], **Ru31–32** [55], **Ru33** [56], **Ru36** [57], and **Ru38** [58] (Figure 6 and Figure 7) altered the mitochondrial function and generated ROS in lung tumor cells, leading to various adverse effects. [*cis*-[Ru(η^2^-O_2_CC_7_H_7_O_2_)(dppm)_2_]PF_6_] (dppm = bis(diphenylphosphino)methane) (**Ru30**), with stability in DMSO monitored by 31P {1H} NMR experiments, showed high cytotoxic activity against Leishmania promastigotes [91,92] and selectively targeted lung target tumor cells [54], while no toxic effect was observed on normal bronchial epithelial BEAS-2B cells. In addition, the increased ROS levels generated by 3.8 µM **Ru30** in A549 lung tumor cells caused oxidative stress, which led to proliferation inhibition, changes in the morphology and organization patterns of the actin cytoskeleton, G2/M phase arrest, apoptosis, changes in the mitochondrial membrane potential, and DNA damage [54]. **Ru31** [Ru(dip)_2_(SA)] and **Ru32** [Ru(dmp)_2_(SA)] (dip = 4,7-diphenyl-1,10-phenanthroline; dmp = 2,9-dimethyl-1,10-phenanthroline; SA = salicylate), two Ru(II) complexes bearing *O*,*O*-chelated ligands with low toxicity to BEAS-2B, could also induce apoptosis in A549 cells via caspase family proteins and PARP activation, ROS accumulation, DNA damage, MMP reduction, and Cytochrome c release from mitochondria [55]. Significant in vitro anticancer activity, combined with proven solution stability and hydrophobic property, prompted the group to further conduct in vivo experiments to test the toxicity [55]. Accordingly, developing zebrafish embryos were used, incubating with various concentrations (0, 12.5, 25, 50, 100, and 200 μM) of **Ru31** in water. Although lower cumulative hatch rate and increased lethality rate, even unhealthy features such as pericardial cysts and spine curvature were notably observed with treatment concentrations up to 200 μM, but there were no apparent side effects found in zebrafish embryos after treatment with concentrations from 12.5 to 50 μM of **Ru31**. Combined with low toxicity towards both normal BEAS-2B cells in vitro and zebrafish embryos in vivo implied that **Ru31** has great potential to develop as a promising therapeutic agent against lung cancer with safety profiles [55].

Caspase-related family proteins are key components of induction and transduction of apoptotic signaling in cells and are closely related to BCL-2 family proteins that either induce (pro-apoptotic) or inhibit (anti-apoptotic) apoptosis [93,94]. **Ru31** downregulated the expression levels of the anti-apoptotic proteins BCL-2 and BCL-xL, whereas it upregulated the levels of the pro-apoptotic proteins BAX and BAD [55]. Since the high expression levels of BCL-2 and BCL-xL have been associated with cisplatin resistance and tumor recurrence in NSCLC patients [95], **Ru31** may indirectly inhibit the resistance and relapse of lung cancer. [Ru(dmp)_2_(pddppn)](ClO_4_)_2_ (pddppn = phenantheno [1,2-b]-1,4-diazabenzo [i] dipyrido [3,2-a:2′,3′-c]phenazine) (**Ru33**) could also downregulate the expression of BCL-2, BCL-x, BAK, and BIM, while upregulating the expression of BAG-1 and BAD, thus inducing apoptosis of NSCLC A549 cells via an intrinsic ROS-mediated mitochondrial dysfunction pathway [56]. Moreover, **Ru33** effectively inhibited the growth of BEL-7402 (human hepatocellular cell line), HeLa (human cervical cancer cell line), MG-63 (human osteosarcoma cell line), and A549 cancer cells with IC_50_ values of 1.6 ± 0.4, 9.0 ± 0.8, 1.5 ± 0.2, and 1.5 ± 0.3 μM, respectively [56]. 

Another study investigated the mechanism of action of Ru complexes, showing that **Ru36** can lead to significant ROS production and induce apoptosis, while increasing the BAX/BCL-2 ratio and PERK levels without affecting the expression of caspase-3 [57]. Four additional Ru(II) complexes with different ancillary ligands { [Ru(bpy)_2_(dmbpy)](PF_6_)_2_ (**Ru34**), [Ru(phen)_2_(dmbpy)](PF_6_)_2_ (**Ru35**), [Ru(bphen)_2_(dmbpy)](PF_6_)_2_ (**Ru36**), [Ru(BPS)_2_dmbpy]Na_2_ (**Ru37**) (dmbpy = dimethyl-2,2′-bipyridine)} (Figure 7) were also prepared in the same study, and their effect on NSCLC (A549) and triple-negative breast (MDA-MB-231) cancer cells were investigated [57]. The increase in lipophilicity as a strategy to enhance the cellular uptake and the antiproliferative activity of the Ruthenium complexes on cancer cells is important [96,97]. Among them, **Ru36** was the only lipophilic complex and exhibited the highest cellular uptake and significant phototoxicity [57]. [Ru(MeIm)_4_(*p*-cpip)]^2+^ (*p*-cpip = 2-(4-chlorophenyl)-1*H*-imidazo [4,5-f] [1,10]phenanthroline, MeIm = 1-methylimidazole) (**Ru38**) has also been recently synthesized and characterized, exhibiting relatively high cytotoxicity against lung cancer (A549) cells, as well as high selectivity to tumor vs. normal cells compared to cisplatin [58]. In addition, **Ru38** induced apoptosis via the mitochondrial pathway, which involved ROS accumulation, mitochondrial dysfunction, and activation of BCL-2 and caspase family proteins [58]. 

### 3.2. Autophagy

Unlike apoptosis, autophagy is an evolutionarily conserved degradation pathway for the recycling of cytoplasmic components and plays a significant role in response to metabolic and therapeutic stresses [98,99]. Recent studies presented scientific evidence supporting the participation of autophagy in tumor progression, such as the development of multidrug resistance of cancer cells, but autophagy also can help in killing cancer cells that are resistant to anticancer agents as a potential target for cancer therapy [99,100].

To the best of our knowledge, there are only a few studies on the inhibition of cell growth caused by metal complexes through autophagy induction. For instance, a Pt-based complex, [Pt(O,O’-acac)(γ-acac)(DMS)], induced autophagy in Caki-1 renal cancer cells [101] and rat B50 neuroblastoma [102]. Apart from Pt-based agents, Piccolo M et al. [103] and Irace C et al. [104] have certified that the activation of autophagic pathways is a common feature with Ru(III) complexes against breast cancer cells. These findings could pave the way for the development of other autophagy-inducing metal complexes to overcome apoptosis-resistant cancer cells. In 2016, the mechanism of Ru(II) complex targeting lung cancer cells by autophagy induction was reported by Chen, L et al. for the first time [59]. Specifically, the formation of autophagosomes and acidic vesicular organelles along with LC3-II upregulation was observed in A549 and NCI-H460 cancer cells treated with a Ru(II) imidazole complex, [Ru(Im)_4_(dppz)]^2+^ (dppz = pyrido [3.2-a:2′,3′-c]phenazine) (**Ru39**) (Figure 7). **Ru39** caused mitochondrial dysfunction and ROS generation in A549 cells, thus partially inducing caspase-3-dependent apoptosis, which causes cell death, as well as autophagy mediated by the extracellular signal-regulated kinase (ERK) signaling pathway. Eventually, the accumulation of **Ru39** in mitochondria could induce autophagy and compete with mitochondria-mediated apoptosis [59]. Furthermore, the antitumor activity of **Ru39** was further evaluated in vivo mice bearing A549 xenografts, which were treated with **Ru39** at 10 or 20 mg/kg. After 28 days, it was observed that the weight and volume of tumors were significantly reduced, and the expressions of LC3-II, cleaved caspase-3, CD-31, and Ki-67 were up-regulated via immunohistochemical analysis. Accordingly, **Ru39** could inhibit tumor growth very well in vitro and in vivo and develop as a promising anticancer candidate promoting cell death via both apoptosis and autophagy pathway [59]. Resembled Ru-based complexes were also highly active against glioblastoma cell lines, inducing cell death via apoptosis and autophagy in a p53-independent manner [105].

### 3.3. Necroptosis

Promoting apoptosis is one of the main methods to treat tumors. However, the anti-apoptotic properties may lead to resistance to cytotoxic chemotherapeutic drugs or radiotherapy [106]. Necroptosis is another type of regulated cell death closely related to the receptor-interacting protein kinases 1 and 3 (RIPK1 and RIPK3) and mixed-lineage kinase domain-like protein (MLKL) [107]. Necroptosis begins with the activation of RIPK and MLKL, which increase the levels of Ca^2+^, causing lysosomal membrane permeabilization, and release cathepsins into the cytosol [108]. Although relevant studies are scarce, a recent report has shown that inducing necroptosis in cancer cells can overcome chemotherapy failure due to apoptotic resistance [109], while metal complexes have been shown to induce cancer cell death through necroptosis [110,111].

**Figure 8:**
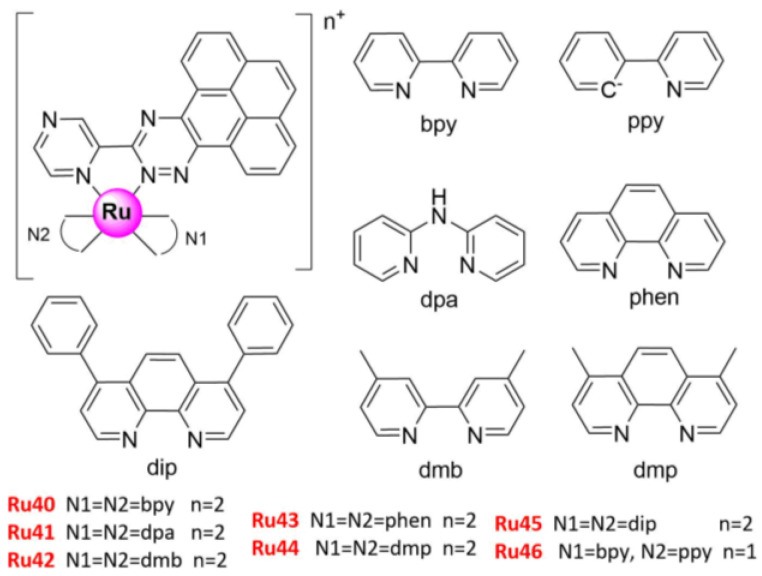
Structures of promising Ru(II) complexes inducing cell necroptosis.

A series of Ru(II) complexes bearing 1,1-(pyrazin-2-yl) pyreno [4,5-e] [1,2,4] triazine with different ancillary ligands have been recently prepared and used to induce necroptosis by Xiong K et al. (**Ru40–46**) (Figure 8) [60]. All analogs showed significant antitumor activity against drug-resistant cancer cells, including A549 lung cancer cells (IC_50_ (**Ru46**) = 3.0 ± 0.1 μM). More importantly, **Ru40–46** prevented the DNA binding of topoisomerases (topo) I and II, which are crucial nuclear enzymes that regulate DNA replication, transcription, recombination, and chromosome segregation during mitosis [60,112]. The characteristic indicators of necroptosis, including ROS burst, plasma membrane permeabilization, and cytosolic ATP reduction, were also reported, while the cell signaling pathway from topo I and II inhibition, DNA damage, and PARP1 activation to necroptosis induced by the activation of RIPK1, RIPK3, and MLKL were elucidated [60]. In contrast, the group also reported a collection of Ru(II) complexes containing asymmetric tridentate ligands that could induce DNA damage and cancer cell death through apoptosis, which were also topo I and II inhibitors [113]. Moreover, caspase-8 has been proven to regulate the switch from apoptosis to necroptosis [114].

### 3.4. Anti-Metastasis

**Figure 9:**
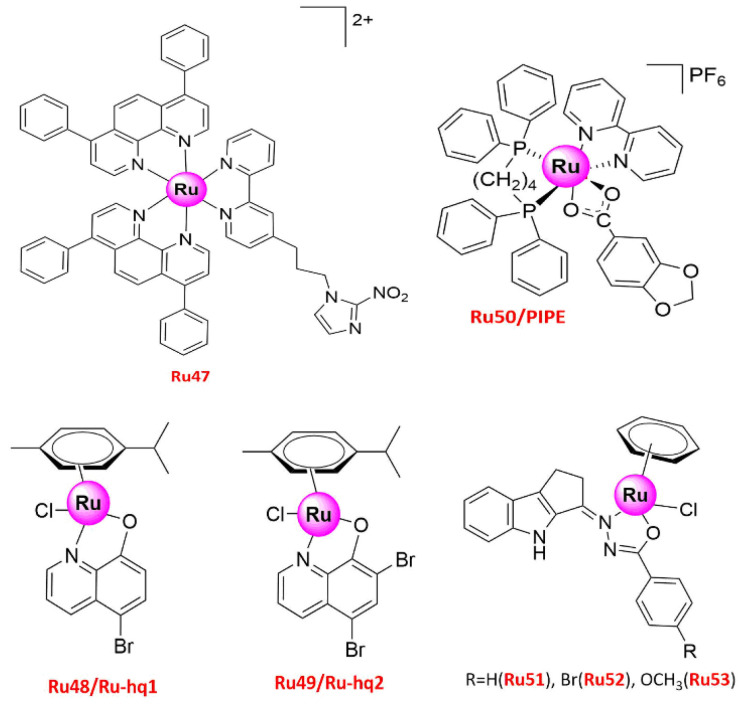
Structures of promising Ru(II) complexes inhibiting tumor metastasis.

Ru(II) compounds can not only suppress the primary tumor but also effectively inhibit malignant tumor metastasis. A series of recently synthesized Ru(II)-containing polypyridyl ligand complexes (**Ru25–29** (Figure 6) [53] and **Ru47** (Figure 9) [61]) showed anti-metastatic properties, which was attributed to their targeting ability to MMPs [53]. **Ru25–29** bearing 2,2′-bipyridine substituted with a semicarbazone-2-formylopyridine moiety as one of the ligands and 4,4′-di-tert-butyl-2,2′-dipyridyl or 4,7-diphenyl-1,10-phenanthroline as auxiliary ligands have been studied for their effect on the adhesion properties of human A549 and pancreatic cancer cells [53]. All complexes enhanced the cell adherent properties and could directly inhibit the activity of MMP2 and MMP9 enzymes in vitro. Among them, **Ru28** led to the most significant enhancement of cell adhesion with increasing concentration [53].

Another Ru polypyridyl complex bearing an nitroimdazole unit {[Ru(dip)_2_(bpy-2-nitroIm)]Cl_2_ (dip = 4,7-diphenyl-1,10-phenanthroline, bpy-2-nitroIm = 4- [3-(2-nitro-1*H*-imidazol-1-yl) propyl]-2,2′-bipyridine)} (**Ru47**) could reduce the activity of MMP enzymes to inhibit tumor metastasis [115], while its effect on cancer and endothelial cells has also been reported [61]. Similar to **Ru25–29**, **Ru47** changed the cell adhesion properties, thus reducing the number of adherent cells on different surfaces (fibronectin, collagen, and plastic) and the expression levels of several MMPs (MMP1a, MMP3, MMP9, LOX, TIMP1, THBS1, and ITB1) and protein-lysine 6-oxidase, while increasing the expression of the extracellular matrix inhibitor [61]. **Ru47** was also studied in hypoxia, as hypoxia results from poor vascular organization of the tumor, promoting drug resistance and malignant progression [116,117]. The results suggested that **Ru47** was more cytotoxic against human breast (4T1) and lung (A549) cancer cells than cisplatin, while it induced cell apoptosis and affected endothelial cell vasculature by activating oxidative stress and modulating the mRNA expression profile of ICAM-1 and VCAM-1 genes involved in metastasis and angiogenesis [61].

Two other complexes, **Ru48/Ru-hq1** [η6-*p*-cymene) Ru(5-bromo-8-hydroxyquinolinato) Cl] and **Ru49/Ru-hq2** [(η6-*p*-cymene) Ru (k2-O,N-5,7-dibromo-HyQ) Cl] (Figure 9), have also been screened as potential novel agents for bone, lung, and breast cancer chemotherapy [62]. Both complexes are stable in DMSO and DMEM solution within the time-frame of the biological experiments and attenuated cell viability with greater selectivity and specificity than cisplatin, while they inhibited cell proliferation, migration, and invasion on cell monolayers at lower concentrations (2.5–10 µM). The higher inhibitory effect of **Ru48/Ru-hq1** and **Ru49/Ru-hq2** on cell invasion compared to cisplatin was further confirm using 3D multicellular spheroid models [62]. Similar results were obtained for **Ru19**, which inhibited cancer cell migration and invasion and had higher safety margins for animals than cisplatin [86]. Therefore, **Ru48–49** and **Ru19** may be used as cisplatin alternatives to manage lung cancer and inhibit tumor metastasis.

### 3.5. Cell Cycle Arrest

The cell cycle, which involves four main phases (G1, S, G2, and M), contributes significantly to the regulation of cell self-renewal and differentiation, while cyclin-dependent kinases (CDKs) and cycle proteins play a key role in the process [118]. Therefore, clinical trials have been performed recently to explore the synergistic effect of CDK inhibitors and different anticancer drugs on various cancer types [119,120]. Additional studies have shown that metallodrugs can target CDKs and cycle proteins to block the cell cycle in different phases. For instance, Ru(II), Rh(III), Mn(II), and Zn(II) complexes blocked the cell cycle in the S-phase, thus inhibiting cell proliferation by reducing the levels of cyclins A2/B1/D1/E1, CDK-2/6, and PCNA and increasing the levels of p21, p27, p53, and CDC25A [121].

**Figure 10:**
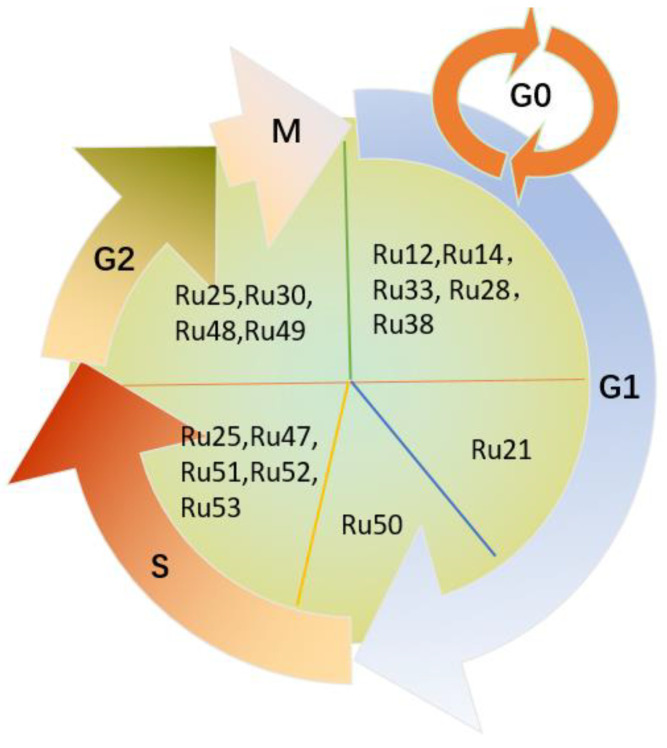
Ru complexes induce cell cycle arrest.

Given that the dysregulation of the cell cycle is associated with the development of cancer, novel Ru(II)-based drugs that inhibited tumor proliferation were synthesized and identified to disrupt the cell cycle in the G0/G1, G1/S, S, and G2/M phases (Figure 10). **Ru12** [82], **Ru14** [85], **Ru33** [56], **Ru28** [52], and **Ru38** [58] are typical examples of compounds causing cell death via cell cycle arrest in the G0/G1 phase. Investigation of the mechanism of action revealed that **Ru12** could induce cell cycle arrest in the G0/G1 phase; suppress the expression of cell-cycle-regulatory proteins, such as cyclins D1, A1, and B1, upregulate p53, p21, and p15; and cleave PARP [82]. Interestingly, the anti-proliferative complex **Ru33** exhibited different mechanisms against different cancer cells: G2/M phase arrest in BEL-7402 and MG-63 cells and G0/G1 phase arrest in A549 cells [56]. In addition, **Ru28** promoted cell accumulation in the G0/G1 phase, while the number of **Ru25**-treated A549 cells increased remarkably in the S-phase but decreased in the G2/M phase [52]. Furthermore, the Ru(II) methylimidazole complex **Ru21** inhibited the growth of A549 lung cells by inducing apoptotic cell death, as confirmed by the generation of a significant apoptosis peak in the sub-G1 phase [88].

The cytotoxicity of {[Ru(pipe)(dppb)(bipy)]PF_6_} (pipe = piperonylic acid, dppb = 1,4-bis(diphenylphosphino) butane) (**Ru50**) on A549 cells has been associated with the induction of cell apoptosis via the intrinsic pathway [63]. The number of cells was significantly increased in the G0/G1 phase of the cell cycle, while the population of A549 cells in the S-phase was reduced when 9 μM of **Ru50** was used. These results indicated that the G1/S transition depended on the antineoplastic ability of **Ru50**, which reduced the cyclin D1 expression levels and attenuated ERK phosphorylation [63]. Furthermore, Ru(II)-based compounds, such as the polypyridyl Ru complex **Ru47** [61], arrested cell growth in the S-phase. Similar results were also obtained for the arene Ru(II) carbazole-based hydrazone complexes **Ru51–53** [(η^6^-benzene) Ru(L) Cl] (L = carbazolone benzhydrazone ligands) [64] (Figure 9), whereas **Ru30** [54] and **Ru48–49** [62] induced apoptosis and cell cycle arrest of A549 lung cancer cells in the G2/M phase.

## 4. Conclusions/Discussions

Lung cancer remains a life-threatening malignancy due to poor prognosis and drug resistance, which is one of the most severe challenges that still need to be addressed to improve patients’ prognosis and survival rate. However, specific drugs preventing tumor metastasis and recurrence have not yet been efficiently developed. In order to design and synthesize effective anticancer agents, transition metal-based compounds have gradually evolved as promising drug candidates due to their cytotoxicity and ability to prevent drug resistance in tumor cells. Among them, Ru(II/III)-based compounds proved to be the most effective, as they show high cytotoxicity, which induces apoptosis, necroptosis, or autophagy and cell cycle arrest, thus inhibiting cell proliferation, invasion, and metastasis (Figure 2 and Figure 10). Most studies suggested that Ruthenium complexes were low in toxicity, easily absorbed, and excreted quickly. More importantly, Ruthenium complexes were easily absorbed by tumor tissues.

In this review, we summarized the recent developments of Ru(II) and Ru(III) complexes and discussed their biological activity and mechanism of action against lung cancer. The existing findings clearly support that Ru complexes can be used to develop effective chemotherapeutic agents for human lung cancer, while they may serve as a guide for the design of other metallodrugs with higher efficiency and better clinical application potential. Nevertheless, the anticancer mechanisms of Ru(II/III) complexes require further investigation, and their efficiency against other cancer types should also be explored in future studies.

## Figures and Tables

**Table 1:** Ruthenium complexes as promising candidates against lung cancer.

No.	IC_50_ (μ/M)	Cell Lines	Biology and Mechanism	Ref.
Ru1	10–12.5 ± 0.5	H1299	Cytotoxicity	[48]
Ru2	15–20 ± 0.5	H1299		
Ru3	30 ± 5	A549	(1) Anti-proliferation (2) Pro-apoptosis (3) Caspase 3/7-dependent apoptosis	[49]
Ru4	5 ± 2.6	A549		
Ru5	18 ± 0.67	A549	(1) Anti-proliferation (2) Pro-apoptosis (3) Enhanced the LDH, NO, and ROS release	[50]
Ru6	24 ± 1.0	A549		
Ru7	22 ± 1.17	A549		
Ru8	24 ± 0.93	A549		
Ru9/G26b Ru10/G94a	>200	A549	(1) Anti-proliferation (2) Anti-metastasis (3) Suppressed MMP-2, MMP-9 and VEGF	[51]
Ru11/AH197	5.0	HOP-62	Anti-metastasis and inhibited cell motility	[51]
Ru12	0–20 0–20	A549 A427	(1) Anti-proliferation (2) Pro-apoptosis (3) G0/G1 phase arrest (4) DNA damage, caspase-dependent apoptosis involving PARP activation and induction of p53-dependent pathway	[51]
Ru13	-	A549	(1) Anti-proliferation	[51]
Ru14	30.9	A549	(1) Anti-proliferation (2) G0/G1 phase arrest (3) induced late apoptosis	[51]
Ru15–18	-	A549	(1) Anti-proliferation	[51]
Ru19	1.39 ± 0.15 3.39 ± 0.47	A549 A549cisR	(1) Anti-proliferation (2) Induced apoptosis (3) Anti-migration and anti-invasion	[51]
Ru20	1.41 ± 0.23 5.70 ± 0.33	A549 A549cisR	(1) Anti-proliferation (2) Induced apoptosis	[51]
Ru21	17.34 ± 0.42 51.59 ± 2.57	A549 HBE	(1) Anti-proliferation (2) Pro-apoptosis (3) Generated a peak of apoptosis in sub-G1 phase, via both mitochondrial and death receptor apoptotic pathways	[51]
Ru22	21.97 ± 2.31	A549	Anti-proliferation	[51]
Ru23	444.38 ± 3.19	A549		
Ru24	37.62 ± 2.83	A549		
Ru25	>240	A549	(1) Anti-proliferation (2) Apoptosis induction (3) S-phase arrest, G2/M phase arrest (4) Anti-metastasis (5) Inhibited MMP2 and MMP9 enzyme activities, intrinsic mitochondrial pathway-triggered apoptosis	[52]
Ru26	158 ± 15	A549	(1) Anti-proliferation (2) Anti-metastasis (3) Inhibited MMP2 and MMP9 enzyme activities	[53]
Ru27	14.1 ± 0.3	A549		
Ru28	10.7 ± 0.7	A549	(1) Anti-proliferation (2) Anti-metastasis (3) G0/G1 phase arrest (4) Pro-apoptosis (5) Inhibited MMP2 and MMP9 enzyme activities, caspase-independent apoptosis	[52]
Ru29	>240	A549	(1) Anti-proliferation (2) Anti-metastasis (3) Inhibited MMP2 and MMP9 enzyme activities	[53]
Ru30	3.8 (2.3–6.2) 40.3 (22.6–71.7)	A549 BEAS-2B	(1) Anti-proliferation (2) G2/M phase arrest (3) The changes in morphology and organization patterns of the actin cytoskeleton, apoptosis, mitochondrial membrane potential changes and DNA damage result from increased ROS	[54]
Ru31	11.3 ± 1.1 54.3 ± 3.4	A549 BEAS-2B	(1) Anti-proliferation (2) Induced apoptosis (3) Through Mitochondrial Apoptotic Pathway, and ROS accumulation, DNA damage	[55]
Ru32	30.1 ± 1.2 48.1 ± 3.7	A549 BEAS-2B		
Ru33	1.5 ± 0.3	A549	(1) Anti-proliferation (2) Induced apoptosis (3) G0/G1 phase arrest (4) Through an intrinsic ROS-mediated mitochondrial dysfunction pathway	[56]
Ru34	12.94 ± 0.43 (Dark) 13.84 ± 3.57 (Light)	A549	(1) Anti-proliferation	[57]
Ru35	17.42 ± 2.39 (Dark) 2.90 ± 0.83 (Light)	A549		
Ru36	6.03 ± 0.89 (Dark) 1.25 ± 0.17 (Light)	A549	(1) Anti-proliferation (2) Phototoxicity (3) Induced apoptosis (4) ROS production and increased Bax/Bcl2 ratio and PERK levels	[57]
Ru37	89.30 ± 3.95 (Dark) 21.89 ± 4.53 (Light)	A549	(1) Anti-proliferation	[57]
Ru38	18.3 ± 2.7	A549	(1) Anti-proliferation (2) Induced apoptosis (3) G0/G1 phase arrest (4) Via the mitochondrial pathway, ROS accumulation, the mitochondrial dysfunction and Bcl-2 and caspase correlative family member activation	[58]
Ru39	21.24 ± 1.24 23.10 ± 3.2 176.47 ± 13.4	A549 NCI-H460 HBE	(1) Anti-proliferation (2) Apoptosis and autophagy induction (3) Mitochondrial dysfunction, ROS generation, caspase 3-dependent apoptosis, ERK mediated-autophagy	[59]
Ru46	3.0 ± 0.1	A549	(1) topo I and II inhibitors (2) induced necroptosis, via ROS burst, plasma membrane permeabilization, and cytosolic ATP reduction (3) induced DNA damage, activated PARP1, RIPK1, RIPK3, and MLKL	[60]
Ru47	Normoxia 17.5 ± 5.7 (24 h) 3.4 ± 0.5 (48 h) Hypoxia 10.9 ± 2.8 (24 h) 4.9 ± 1.6 (48 h)	A549	(1) Anti-proliferation (2) Anti-metastasis, Anti-invasion (3) Pro-apoptosis (4) S-phase arrest (5) Decreased the number of adherent cells to different surfaces (fibronectin, collagen, plastic) and the expression of several MMPs and protein-lysine 6-oxidase, increased the expression of the extracellular matrix inhibitor	[61]
Ru48/Ru-hq1	50.9 ± 5.3 (2D) 103.9 ± 10.8 (3D)	A549	(1) Anti-proliferation (2) Anti-migration, anti-invasion (3) Pro-apoptosis (4) G2/M phase arrest	[62]
Ru49/Ru-hq2	24.9 ± 6.5 (2D) 213.6.9 ± 8.6 (2D)	A549		
Ru50/PIPE	17.99 ± 0.39 (24 h) 4.11 ± 0.27 (48 h)	A549	(1) Anti-proliferation (2) Induced apoptosis (3) G1/S phase arrest (4) Reduced cyclin D1 expression and ERK phosphorylation levels, and induced apoptosis by intrinsic pathway	[63]
Ru51	10.0 ± 1.0	A549	(1) Anti-proliferation (2) S-phase arrest	[64]
Ru52	7.0 ± 0.5	A549		
Ru53	3.0 ± 0.5	A549		

## Abbreviations

Ru: Ruthenium; NSCLC: non-small-cell lung cancer; SCLC: small cell lung cancer; LUSC: lung squamous cell carcinoma; LUAD: lung adenocarcinoma; BSA: bovine serum albumin; PDT: photodynamic therapy; PACT: photoactivated chemotherapy; MRP1: multidrug resistance-associated protein 1; Pgp: P-glycoprotein 1; RIPK: receptor-interacting protein kinase; MLKL: mixed lineage kinase domain-like protein; Topo: topoisomerase; MCS: multicellular spheroids; CDK: cyclin-dependent kinase; PARP: Poly (ADP-ribose) polymerase protein; ERK: extracellular signal-regulated kinase; EAC: Ehrlich ascites carcinoma; ROS: reactive oxygen species; LLC: lung cancer Lewis; VEGF: vascular endothelial growth factor; AVOs: acidic vesicular organelles; Pt: platinum; MMPs: matrix metalloproteinases.
